# Parental perspectives regarding the return of genomic research results in neurodevelopmental disorders in South Africa: anticipated impact and preferences

**DOI:** 10.1007/s12687-024-00723-w

**Published:** 2024-08-02

**Authors:** Angelique Diedericks, Zandré Bruwer, Nakita Laing, Emma Eastman, Jantina De Vries, Charles R. Newton, Amina Abubakar, Elise B. Robinson, Kirsten A. Donald, on behalf of the NeuroDev study

**Affiliations:** 1 Human Genetics, Department of Medicine, University of Cape Town, Groote Schuur Hospital, Observatory, Cape Town, South Africa; 2 Department of Paediatrics & Child Health, Red Cross War Memorial Children’s Hospital and University of Cape Town, Cape Town, South Africa; 3 The Ethics Lab, Neuroscience Institute and Department of Medicine, University of Cape Town, Rondebosch, Cape Town, South Africa; 4 Neuroscience Unit, Center for Geographic Medicine Research Coast, KEMRI-Wellcome Trust, Kilifi, Kenya; 5 Department of Psychiatry, University of Oxford, London, UK; 6 Institute of Human Development, Aga Khan University, Nairobi, Kenya; 7 The Broad Institute of MIT and Harvard, Cambridge, MA USA; 8 Center for Genomic Medicine, Massachusetts General Hospital, Boston, MA USA; 9 Neuroscience Institute, University of Cape Town, Groote Schuur Hospital, Observatory, Cape Town, South Africa

**Keywords:** Preferences, Research findings, Genomic results, Neurodevelopmental disorders, Parental perspective, Feedback

## Abstract

Few policies and little research exist regarding the disclosure of genomic results to research participants in Africa. As understanding participant preferences would be pivotal to the success of the feedback process, this study set out to address this issue by engaging with enrolled participants from an ongoing genomics research project on neurodevelopmental disorders with the aim to assess the anticipated impact of receiving pertinent results and explore the preferences for feedback in a South African context. Twelve semi-structured interviews were conducted with 17 parents of children participating in the research study. Transcribed interview data and observational notes were analysed using thematic analysis and framework matrices. Participants linked their own meaning to the impact of receiving a pertinent result and perceived the information as useful for reasons other than only clinical utility. These included closure, improved management of their child’s condition and information regarding recurrence risks. In terms of preferences for feedback, an in-person result delivery session, conducted by a member of the study team or medical professional familiar with their child was preferred. In addition, participants felt a sense of ownership over their blood or their contribution to the research study, finding meaning even in non-pertinent results. These findings provide insight into the type of discussions that may be valuable in enabling the development of best practices and guidelines for the return of individual genetic research results, in a culturally appropriate manner, within South African communities.

**Supplementary information:**

The online version contains supplementary material available at 10.1007/s12687-024-00723-w.

## Introduction

One pertinent challenge in genomics research relates to questions about what to do with individual genomic research results that are relevant to the health of the participant. Deciding whether to disclose individual genomic research results, and the researcher’s obligations in this regard, has been the topic of much controversy nationally and internationally, particularly where minors are concerned (Canadian Institutes of Health Research, Natural Sciences and Engineering Research Council of Canada, and Social Sciences and Humanities Research Council of Canada 2018; Council for International Organizations of Medical Sciences 2016; Eckstein et al. 2014; Green et al. 2013; Government of Canada 2019; Miller et al. 2023). With the advent of exome and genome sequencing technologies, the identification of genetic information that is relevant to the health of the individual is inevitable. Initially, there was much debate in genomics research about what findings to return and when. However, consensus now exists that pertinent findings, undiagnosed without genetic research, and supported by sufficient evidence of pathogenicity should be fed back, especially if participants have indicated their willingness to receive such results (Eckstein et al. 2014). This view is also the case in the African genomics research community (Matimba et al. 2022; Nabukenya et al. 2023; Wonkam and De Vries 2020).

In Africa, the Human Heredity and Health in Africa (H3Africa) Consortium facilitates research into diseases in the country while developing infrastructure, resources, training and ethical guidelines to support African research led by African scientists (Matimba et al. 2022). The H3Africa Ethics and Community Engagement working group developed a set of guiding principles to inform decisions about returning individual genetic results to research participants. Emphasis is placed on the ethical obligation to return pertinent findings (findings related to the disease or condition being investigated by the study) with important health implications for the participant and which have the potential to be medically actionable in relation to the population being investigated (Matimba et al. 2022). Other considerations also include the analytic utility, the personal utility of the finding to the participant, and whether participants have consented for the return of results (Matimba et al. 2022; Rotimi et al. 2014). However, for secondary findings, the return of this information is not considered obligatory by the H3Africa Consortium due to the weak evidence base for African populations. This includes the underrepresentation of African ancestry in clinical and research databases and specific disorders within the ACMG actionable genes list (Matimba et al. 2022).

Contextualization in disclosing genomic research results remains fundamental, even more so in a community like South Africa that is linguistically and culturally diverse (Munung et al 2016; Nembaware et al. 2019; Tekola et al. 2009). Additionally, the socio-economic context of Africa, characterized by poverty and under-resourced healthcare systems, further complicates access to healthcare (Matimba et al. 2022; Wonkam and de Vries 2020). With the exception of the H3Africa feedback of individual genetic research findings guidelines, few other policies and little research exists regarding the disclosure of genetic results to research participants in Africa (African Academy of Sciences 2021; Matshabane et al. 2020; Wonkam and de Vries 2020). As understanding participant preferences is pivotal to the success of the feedback process, this work set out to address the issue by engaging with enrolled participants from a genomics research study conducted through the NeuroDev project, to (1) asses the anticipated impact of receiving pertinent results and (2) explore the preferences for feedback in the South African context. The NeuroDev study is a large-scale, international collaborative project aimed at mapping genetic variation amongst children with neurodevelopmental disorders (NDD) by performing in-depth phenotyping and exome sequencing on children aged 2 – 17 years old from populations in South Africa and Kenya (de Menil et al. 2019; Kipkemoi et al. 2023). NDDs are a group of complex conditions characterised by developmental deficits such as impaired functioning on a social, personal, academic or occupational level. These conditions typically manifest in early development and according to the Diagnostic and Statistical Manual of Mental Disorders (DSM-V), can include conditions such as autism spectrum disorders (ASD), attention-deficit hyperactivity disorders (ADHD), intellectual disability (ID), global developmental delay (GDD), communication disorders and specific learning disorders (such as dyslexia) (American Psychiatric Association 2013). In keeping with the H3Africa guidelines, the NeuroDev study only returns clinically actionable results associated with NDDs. Variants of uncertain significance (VUS), incidental and secondary findings are not returned. The feedback process is planned to include several steps, namely: re-contacting the participant’s family after identifying a causative genetic variant within the context of the research study, reconsenting, and testing a second sample in a clinical diagnostic laboratory for confirmation, followed by a result-delivery session. This study specifically sets out to investigate preferences for feedback of genomic research results from South African parents of an affected child with a NDD enrolled in the NeuroDev study, ahead of the clinical confirmation phase. Therefore, any important findings or recommendations can be used to inform the next phase of the broader NeuroDev study, namely the delivery of individual genomic results.

## Method

### Study design

A qualitative study was conducted using semi-structured interviews with participants enrolled in the NeuroDev study, to investigate the anticipated impact and parental preferences regarding the return of research results.

### Recruitment and samples

Purposive sampling was used to recruit parents of children who participated in the NeuroDev study and had consented to receiving pertinent research results. Eligibility criteria for the qualitative study required parents to be: 1) the biological parent or primary caregiver of a participant in the NeuroDev study 2) illustrate cognitive capacity to consent and 3) have consented in the NeuroDev study to be contacted for secondary/any further research.

Participants were contacted telephonically by one of the authors and informed about the aims and objectives of the study. If they were interested in participating, a date and time was set-up for an in-person interview. Twelve semi-structured interviews were conducted with 17 participants in a private room at the Red Cross War Memorial Children’s Hospital in Cape Town South Africa from May to July 2019. Importantly, whilst all participants were parents of children recruited into NeuroDev, no results from the exome sequencing component of the study were yet available and none of the parents had yet received research results. At this stage it was not yet evident which parents would receive individual genetic results directly relevant to the condition of their child.

### Procedures

The interview guide was developed based on a review of the literature and examination of any previously developed guides from similar research conducted in other settings (Allen et al. 2014; Harris et al. 2012; Holm et al. 2015; Hylind et al. 2018; Munung et al. 2016; Sapp et al. 2014; Traore et al 2015). The questions were structured around the following topics: anticipated impact of pertinent genetic research results and preference for the feedback of findings. Questions were adapted to suit the South African setting and align them with the study’s objectives. Pilot interviews were performed to explore the utility and structure of the interview questions. While South Africa has eleven official languages, those that are dominant in the Western Cape include English, Afrikaans and isiXhosa (Census 2022; Western Cape Language Audit 2001). Therefore, translation services were offered to all participants however they declined this, and all interviews were subsequently conducted in English by the first author. Interviews lasted approximately one hour and were audio-recorded. Field notes were written immediately following interviews and captured any elements or observations relevant to the research and data analysis. Transcription was done by a dedicated transcription company and the research team reviewed the transcripts for accuracy. The interview guide appears in S1.

### Data analysis

Interview data and observational notes were analysed using thematic analysis and framework matrices (Gale et al. 2013). Themes were extracted from the data following transcription of the initial interviews. Through using framework analysis, robustness and rigour was attained by allowing for clear audit trails of cases (Gale et al. 2013; Hackett and Strickland 2019). The research team manually analysed the first three transcripts together, examining the relevance of themes and agreeing on any required re-classifications or modifications. The first author then continued with the coding and discussed any new findings with the rest of the research team, to enable agreements to be reached to prevent potential bias of a single rater. An open coding strategy, which assigned open codes to relevant or interesting text excerpts, was utilized. Drawing on the list of open codes, a mind map was compiled with preliminary themes, after which both themes and coded text were imported into Excel and further reorganized into sub-themes/categories. Transcripts were imported into NVivo 12 to assist with managing and organizing the data for analysis. Emerging insights were recorded in field notes and were further probed in subsequent interviews. To ensure further credibility, quotations from research participants were incorporated into text excerpts which assisted with providing thick descriptions and context of participant meaning and experiences (Korstjens and Moser 2018).

Ethical approval for this study was obtained from the University of Cape Town Human Research Ethics Committee (HREC 784/2018).

## Results

**Fig. 1:**
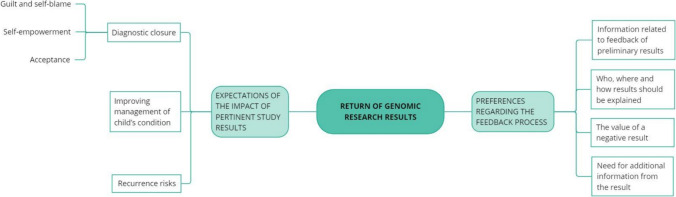
Overview of thematic domains identified from analysis

A total of 17 participants were recruited with both individual and couple interviews conducted with the parents of children enrolled in the NeuroDev study. Socio-demographic data (Table 1) together with a description of emergent themes (Fig. 1) provide further insight into participant expectations and preferences for the return of research results.

**Table 1:** Individual participant characteristics

Demographic characteristics	Individual interview (n = 17)
Gender	
* Female*	12
* Male*	5
Ethnicity/cultural background*	
* Mixed Ancestry (Coloured)*	12
* African Ancestry (Xhosa)*	5
Format of interview	
* Number interviewed individually*	7
* Number interviewed in couples*	5
Description of NDD	
Number of parents with a child with NDD including ASD	15
Number of parents with a child with NDD	2

*self-reported ethnicity/culture

NDD – Neurodevelopmental disorder

ASD – Autism spectrum disorder

## Expectations of the impact of pertinent study results

Broadly speaking, participants recalled consenting to receiving individual genetic research results for their children. The expectation from these results were that they could possibly bring diagnostic closure, help in the management of their child’s condition, or help inform reproductive decisions by clarifying the risk of recurrence in other children.

### Diagnostic closure

Diagnostic closure encompassed acceptance of their child/situation, addressing guilt and blame and self-empowerment in the form of having a name for their child’s condition. These three sub-themes are elaborated on below:

- **Acceptance**

Many participants expressed that whilst the unknown brought fear, results could bring awareness and acceptance. Other participants reported that a genetic answer would bring relief regardless of the meaning of the result or the cause of their child’s condition.

*“Awareness and being able to accept whatever it is and …, it just makes you feel more at peace about whatever, you know, whatever the result is. But not knowing is scary. You don’t know how to deal with things and, I mean, we used to think that [child's name] is just being naughty, you know, and sometimes you feel bad because we used to scold him and give him a spanking whatever, and in the meanwhile he had this problem. So ja, I’d rather be aware and know, and then know how to treat it.” (Participant 16)*

*“It would be a relief. Because now we’re just saying, okay, maybe ja, it’s the vaccine. But maybe it’s not. Maybe there’s something wrong with me and [husband's name]…So you know, if they come and say we found something on [name of child], yo, it will be … it doesn’t matter if it’s bad or wrong but it will be a relief.” (Participant 11)*

- **Guilt and self-blame**

Most participants felt that results would resolve feelings of guilt and self-blame by offering an understanding of why it happened and where it came from. One mother was searching for confirmation that she was not to blame. She felt that she had done everything right during the pregnancy—in fact more so than in a previous pregnancy which resulted in an unaffected child:

*“It’s just a need to know. Because when I, like in the beginning when we found out … the first thing we did we stopped smoking. That’s the first thing we did. So, we did everything right. We didn’t do unnecessary going out. We just took it easy. In fact, I was more healthy with her to what I was with him [sibling] in my pregnancy. So I would like to know how and why.” (Participant 15)*

A few participants expressed concern that internal guilt could be exacerbated if the condition was found to be inherited:

*“… But if it’s going to come like something that he get from me I’m going to feel guilty on the other side, because even when I look at him suffering or it’s difficult to do something I’m going to be like … you are like this because of me” (Participant 7)*

- **Self-empowerment**

Participants hoped that results from genomic testing would provide them with a name for their child’s condition, thereby providing means for defined management and treatment. The participant below noted the benefits of having a name for their child’s condition in terms of allowing them to conduct their own research and foster self-empowerment:

*“… it will also make things easier for research, for me. When I … like when we discovered when she was born this was wrong … it’s easy to Google once you have a name to something. But before they could tell me what was wrong with her eyes I was laying in the bed after giving birth and was trying to Google, born without a right eye, what does it mean. And ten thousand stuff came up and I’m like, now which one of these does she have. So, it would be nice to know.” (Participant 15)s*

### Improving management of child’s condition

For many, becoming as educated as possible about their child’s condition meant that they would have a greater understanding and ability to manage their child:

*“I mean, I just want to be like as educated as I can on the subject. Because I mean, it’s part of my life now.” (Participant 1)*

*“I think it’s going to teach me something also so that I can know how to handle him, all that. Because if you know what is wrong with this person then you know what to do when you are around this person.” (Participant 7)*

Participants further pointed out that having results could make things easier, raise hopes for treatment or medicines and an improved life for their child. One parent expressed that:

*“Like maybe what medication he can use, or something. I’ve seen a video of a … I think the girl is 24 and she’s got autism, can speak and everything. She’s got her own school for autistic children that actually helps them, but it’s not here. It’s overseas. Basically just to help him to improve his life.” (Participant 6)*

### Recurrence risks

Receiving pertinent results could inform participants of their personal reproductive potential:

*“I remember like the main reason why we did it, was so that we could find out if we could have more children without the child having some type of neurological issue” (Participant 1)*

Or that of their children:

*“So I said I understand. I don’t mind no matter it’s not going to help me now, maybe it’s going to help my grandchild in the future.” (Participant 7)*

## Preferences regarding the feedback process

### Information related to feedback of preliminary results

The participant’s need for information varied. Almost half preferred to only be told of the result after clinical confirmation. The most prominent reasons for waiting for the final result included avoiding unnecessary stress and worry over an inconclusive result, the need for certainty and to maintain peace of mind.

*“Because I want like solid evidence that this is what they’ve found. I don’t like this could be and that could be and then take the second sample and then it’s like a totally different ballgame and that’s why I’d prefer end results and in fact we’re willing to wait longer for that.” (Participant 1)*

A few expressed their desire for receiving preliminary research results, indicating a need for study involvement and to be prepared for the possible outcome.

*“I would like to know that yes. Like what is it and why? What did you pick up that you just want to verify. Yes, I know maybe it’s not it, but just so I know exactly where they are heading.” (Participant 15)*

Further reasons included wanting to know if they needed to be concerned, the desire to assist in the research being conducted but expecting researcher transparency throughout, the right to know and be informed.

*“It’s also the need to understand how they got to the point. So yes, they found something they’re not sure and they need to double check it. But it would be nice to know that you guys actually … there’s some type of progress. Because it’s going somewhere and not just, we’re back here again.” (Participant 15)*

There was a perceived potential for preliminary research results to cure or treat their child, possibly referring to the timeous receipt of results and early intervention.

*“In case there’s something maybe we can fix.” (Participant 14)*

Most participants would automatically assume that a causative variant was found if they were contacted about the collection of a second sample:

*“Obviously I would know then that it’s got something to do with our genetics. So I’d probably wouldn’t want to know because then I’m going to mull over it for the next couple of months while they do the second sample. So I would just assume that something is up with one of us and that they will have to verify it. So I’d prefer to get like actual information rather than this could be and that could be and we’re still busy with that.” (Participant 1)*

### Who, where and how results should be explained

While a few participants felt that they had no preference concerning the person who returned their results, the majority preferred the news to come from the original study researchers or doctors managing/treating their child:

*“Yes. Or even the doctor because this child is going to Dr [name]. Even the doctor explain us it will be fine. I would be happy with that. Because the doctor knows everything of the child.” (Participant 13)*

When participants were asked where they would like to receive feedback regarding their results, consensus was that they wanted to be told at the hospital where their child was attending and during an in-person session. For some, a negative result could be communicated telephonically but it was expressed that a face-to-face encounter, even with negative results, would alleviate anxiety and worry. A few participants additionally stated a distrust of technology and fear of breach of security if telephonic delivery was undertaken:

*“The lines are never secured. You can’t say something is secured because even computers, cyber security, that’s big.” (Participant 10)*

### The value of a negative result

The majority of participants wanted to be contacted with a negative result for various reasons, including getting closure, for peace of mind, keeping calm, and to stay informed of research progress.

*“Yes. I think that is as important. I think it’s more for peace of mind and also to … it’s just about us being included. Keep updated. A negative result is still a result.” (Participant 12)*

For some individuals, receiving a negative result would bring reassurance that they and the researchers did the best they could, despite a lack of answers over what the meaning of the result may be or the lack of clinical utility.

*“Just to let this whole thing come to fruition, I mean like, just have it being done and we … not that I would feel like this has all been for nothing. I mean, I’ve been educated about some subjects with regards to this, and I know what you do. And, ja I just think that I would be okay with even if they didn’t know anything, then at least I know that it’s over and that they’ve done all they could and that it’s still a mystery.” (Participant 1)*

A sense of ownership over their blood and thus their results was also apparent:

*“I prefer they must call me to tell me that. Because I’m going to sit like now, that year in Red Cross they take my blood and they say I didn’t know what they’re checking, I didn’t know if there’s anything that they found in my blood, but that is quiet. Nothing happen after that. I would like to … because it’s my blood, I would like to contact me and tell me even if they didn’t find anything.” (Participant 7)*

Whilst many understood that researcher constraints, such as budget restrictions and the large number of participants enrolled in the study, may not allow for the feedback of negative results, they expressed their appreciation for such feedback.

*“Yes, I would appreciate that. I do know however that there is a budget for all of this and that, I mean, there’s probably hundreds if not thousands of people enrolled in this thing, so it’s not imperative that they have to call if they don’t find a result. But if they are able to do so then, yes, I would appreciate getting a phone call.” (Participant 16)*

### Need for additional information from the result

Whilst participants reiterated that they were aware that only pertinent results would be returned, they continued to discuss the likely implications of non-causative or uncertain results. Many participants expressed their wish to receive all types of results, good or bad, including individual results that would be of significance to the health of their child (secondary findings):

*“If it’s something that can affect him, then yes, we would want to know about it…And they pick up something else that’s not related, yes.” (Participant 5)*

A few parents believed results would be indicative of their own personal health, also referring to secondary findings:

*“Any. I can’t say I’m hoping for this, but any result that comes. Anything that they can find maybe some sicknesses in me or something maybe can … so that they can prevent it to other people…But if they can say the result is coming like this, this is something that we found that may cause your child to be like this, then I will accept it. The moment that they come with the result that says this is a condition we found but it’s not what caused this on your child. That will be fine too. And then I’m going to look forward to see how they’re going to maybe help it or treat it…Everything they find. Everything.” (Participant 7)*

Others felt it would enable them to provide a better quality of life by being prepared and planning for their family’s future. The latter was also described by a participant in terms of his own health, possibly driven by personal fears of having passed the condition on to his child or related to the value of future planning (as a result of the knowledge gained from a secondary finding):

*“Look, I mean if there is a result there’s a result. Obviously we have to know that. I’ve got dependents, five of them you know, so I would want to … if I’m going to die tomorrow or in a few years’ time I would like to have that quality of life. I don’t just want to leave them behind or whatever” (Participant 17)*

Some participants seemed to prefer to receive information about all kinds of results, including ones that were not pertinent (such as VUS). They expressed the hope that those results could lead to further research that they could participate in.

*“Maybe then someone else would come and try to take that further and then we can be involved in that study.” (Participant 1)*

On the contrary, some were unsure about the usefulness of VUS information and expressed concern over the possibility of discovering new/other information, indicating a preference not to know about VUS results.

*“I don’t know if I would want to know that part, because now you’re going to be sitting with this information and you’re thinking what could this be.” (Participant 16)*

## Discussion

This study assessed the anticipated impact of receiving pertinent results and explored feedback preferences among South African families with a child with a NDD, with the aim of shaping the feedback process for the NeuroDev study. Participants identified that pertinent results had the potential to have a positive impact by bringing acceptance of their child’s condition, dissipate feelings of self-blame or guilt and offer a means of self-empowerment. In addition, participants expected individual results to potentially inform on management and recurrence risks, which is consistent with the literature from other African and international studies (Hall et al. 2015; Hylind et al. 2018; Traore et al. 2015). Determining if the condition was inherited or de novo was also found to be associated with participants wanting to resolve feelings of self-blame. Although participants identified that an inherited cause could lead to anxiety as they might feel personally responsible for their child’s condition, potentially exacerbating feelings of being at fault, it was also noted to have the potential to reduce self-blame by allowing the individual to feel less responsible for the condition given that it was not under their control. According to Faure et al. (2019), genetic information can reduce blame but increase the sense of loss of control and feelings of hopelessness, leading to internalized stigma. Other studies on ASD in Australia and Ghana have highlighted that parents often internalize these feelings, blaming themselves for their child’s condition or feel responsible (Broady et al. 2017; Oti-Boadi et al. 2020). From our study, there was some expectation that the return of results would impact on resolving feelings of guilt and perhaps some self-blame as many participants alluded to seeking confirmation or validation that they were not responsible. Being aware of the impact of receiving results on these feelings (both positive and negative associated outcomes) would be valuable when conducting sessions centred around the feedback of findings.

Participants gave diverse preferences concerning the return of results process; however, overall perspectives were the desire for certainty over the cause and therefore willingness to wait for clinical confirmation. As research results cannot be fed back to participants unless it has been verified in a certified diagnostic laboratory, an additional sample would need to be taken for clinical confirmatory testing. Participants in our study described that they would automatically assume a disease-causing variant had been found at the time of contact for a second sample. Whilst some welcomed the opportunity to prepare for the possibility of a positive result and perceived a potential for preliminary results to offer a chance for early intervention, others preferred to receive no explanation; fearing the potential to create anxiety. Although the latter was only identified in the minority of our families, it was acknowledged that individuals involved in the feedback process might face the challenge of dealing with managing participant anxieties during this preliminary feedback process.

The timing and amount of information conveyed during the process may have a degree of psychosocial impact for some as well as ensuring researcher transparency, ultimately influencing participant trust in researchers or the research enterprise. Our findings further suggest that familiarity with the person delivering the result as well as with a preference for the familiar clinic is important for participants when considering who should communicate results, where it should be done, and who should be involved in the process. An in-person result delivery session, conducted by a member of the NeuroDev team or medical professional familiar with their child was preferred and thought to be able to reduce pre-existing anxiety and alleviate concerns for confidentiality. This finding aligns with the H3Africa recommendations, advising that the result delivery session should be undertaken by the clinician or qualified health professionals involved in the genomic research project until other staff are sufficiently trained to take over this task (Matimba et al. 2022).

Participants felt a sense of ownership over their blood or their contribution to the research study, finding meaning even in non-pertinent or negative results. Although all participants were aware that only pertinent results would be returned, they mentioned the value that they still placed on the non-pertinent findings and negative results as they perceived it as a result which could provide answers to issues relating to future health, for themselves or their child, or offer a chance to be involved in forthcoming research studies. These findings resonate with results of other African and South African studies considering the parental preferences and expectations of feedback of findings from genomic research studies (Matshabane et al. 2020; Ralefala et al. 2021, 2022). The authors identified that participants expressed a strong interest in receiving secondary results as they were viewed as valuable because they could empower or emancipate individuals or motivate healthier lifestyle choices. While other international studies identified that participants found meaning in results of uncertainty and believed that knowledge about such results and analysis would grow, yielding answers in the future outcomes (Halverson et al. 2016; Miller et al. 2008). It may be important to consider the anticipated value and meaning participants attribute to results in addition to assessments of clinical utility or medical actionability, as participants may classify these concepts differently to researchers (Holm et al. 2015). Participants in our study clearly described that negative results would also be a valuable return for their participation in the NeuroDev study, albeit the lack of an answer as to the cause of their child’s condition. They viewed such a result as an answer, with the potential to bring closure, peace and acceptance, perhaps through the knowledge of having done all they could. Reasons for wanting all generated results stemmed from a need to plan for the future, to provide quality of life, to inform personal health risks and to promote future research and information discovery. This finding was communicated back to the NeuroDev study. After receiving approval from the Institutional Review Board and Research Ethics Committee, the NeuroDev policy was updated to include the return of negative findings to participants.

### Limitations

Whilst our study has shed some insight into what South African NeuroDev participants would like to know, the sample was small and narrowly tiered for a particular set of conditions. Data may additionally be skewed by the fact that purposive sampling was used whereby the views of those who could not be reached or who declined were not represented. As a result, our findings may not be generalizable to other communities/ethnic groups or regions within South African or countries across the African continent. Sociodemographic data beyond gender and ethnic background was not obtained, although these could have had important influences on participant’s coping mechanisms.

## Conclusion

There is limited literature that documents the views of African families with regards to the expectations for the return of individual genetic findings in genomics research. Our study provides evidence on the views of South African participants with regards to expectations and preference for return of results. It will be important to consider these findings in the feedback process, as well as the importance that these findings may have with regards to discussions around the development of best practices for African-specific guidelines which are contextually relevant. These may include an in-person result delivery session conducted by a member of the study team or familiar healthcare professional, feeding back negative results as well as consideration for a tiered-consent model to enable feedback of secondary findings for participants who would like to receive these.

## Supplementary information

Below is the link to the electronic supplementary material.

Supplementary file1 (DOCX 16 KB)

## Data Availability

The data that supports the findings of this study are available upon request from the corresponding author.

## Declarations

Ethical approval for the study was obtained from the UCT HREC (HREC 784/2018) as well as Institutional approval from Red Cross War Memorial Children’s Hospital.
