# Supplementary material for: Parental perspectives regarding the return of genomic research results in neurodevelopmental disorders in South Africa: anticipated impact and preferences
Source: J Community Genet. 2024 Aug 2;15(5):539–48. doi: 10.1007/s12687-024-00723-w (PMC11549274; doi:10.1007/s12687-024-00723-w)
Supplement: Supplementary file 1 — Supplementary file1 (DOCX 16 KB) [file 12687_2024_723_MOESM1_ESM.docx]

**Appendix S1** Excerpt of interview guide

| 1. | What do you believe to be the reason for your child’s condition?  What is it like living with a child with an undiagnosed condition? |
| --- | --- |
| 2. | Why did you choose to participate in this study?  Do you remember what kind of results they said you can receive? (PROMPT: Only Neurodevelopmental-related/only if they know what the result means for their child/even if they don’t know what the result means)  What kind of results would you like to receive (PROMPT: pertinent, secondary, uncertain)?  What kind of result are you expecting?  What would it mean for you and your family to receive a result? (PROMPT: diagnostic closure, gendered blame, stigma, others?) |
| 3. | What do you remember from the time when they said they will give you a result. ( PROMPT: after 2nd sample is taken and verified)  When would you want to hear about possible results – before the 2nd sample is taken or only after validation?  What are your reasons for wanting/not wanting the results before verification?  If we contact you, how should we do this? (PROMPT: phone call, hospital)  What would you like to know when we take the second sample?  Would you want to be contacted with a negative result? |
| 4. | If we have a verified result, where should we give this result to you and how? (PROMPT: At Red Cross Hospital, at home, phone or Skype)  Who do you feel should be involved?  Who would be your support/who would you talk to regarding the result? |
| 5. | Is there anything else you would want to share with me that I haven’t asked about? |
